# Supplementary material for: Completing the TRB family: newly characterized members show ancient evolutionary origins and distinct localization, yet similar interactions
Source: Plant Mol Biol. 2023 Apr 28;112(1-2):61–83. doi: 10.1007/s11103-023-01348-2 (PMC10167121; doi:10.1007/s11103-023-01348-2)
Supplement: Supplementary file 1 — Supplementary file1 (PDF 1222 KB) [file 11103_2023_1348_MOESM1_ESM.pdf]

Completing TRB family: newly characterized members show ancient evolutionary origins and distinct localization, yet similar interactions

Alžbeta Kusová[1,2+], Lenka Steinbachová[3+], Tereza Přerovská[1], Lenka Záveská Drábková[3], Jan Paleček[1,2], Ahamed Khan[4], Gabriela Rigóová[1], Zuzana Gadiou[3], Claire Jourdain[5], Tino Stricker[5], Daniel Schubert[5], David Honys[3,6], and Petra Procházková Schrumpfová[1,2*]

[1] Laboratory of Functional Genomics and Proteomics, National Centre for Biomolecular Research, Faculty of Science, Masaryk University, Brno, Czech Republic
[2] Mendel Centre for Plant Genomics and Proteomics, Central European Institute of Technology, Masaryk University, Brno, Czech Republic
[3] Laboratory of Pollen Biology, Institute of Experimental Botany of the Czech Academy of Sciences, Prague, Czech Republic
[4] Biology Centre of the Czech Academy of Sciences, Institute of Plant Molecular Biology, České Budějovice, Czech Republic
[5] Institute of Biology, Freie Universität, 14195 Berlin, Germany
[6] Department of Experimental Plant Biology, Faculty of Science, Charles University, Prague, Czech Republic

* Author for correspondence: schpetra@sci.muni.cz
+ Co-authors: Shared co-first authorship.

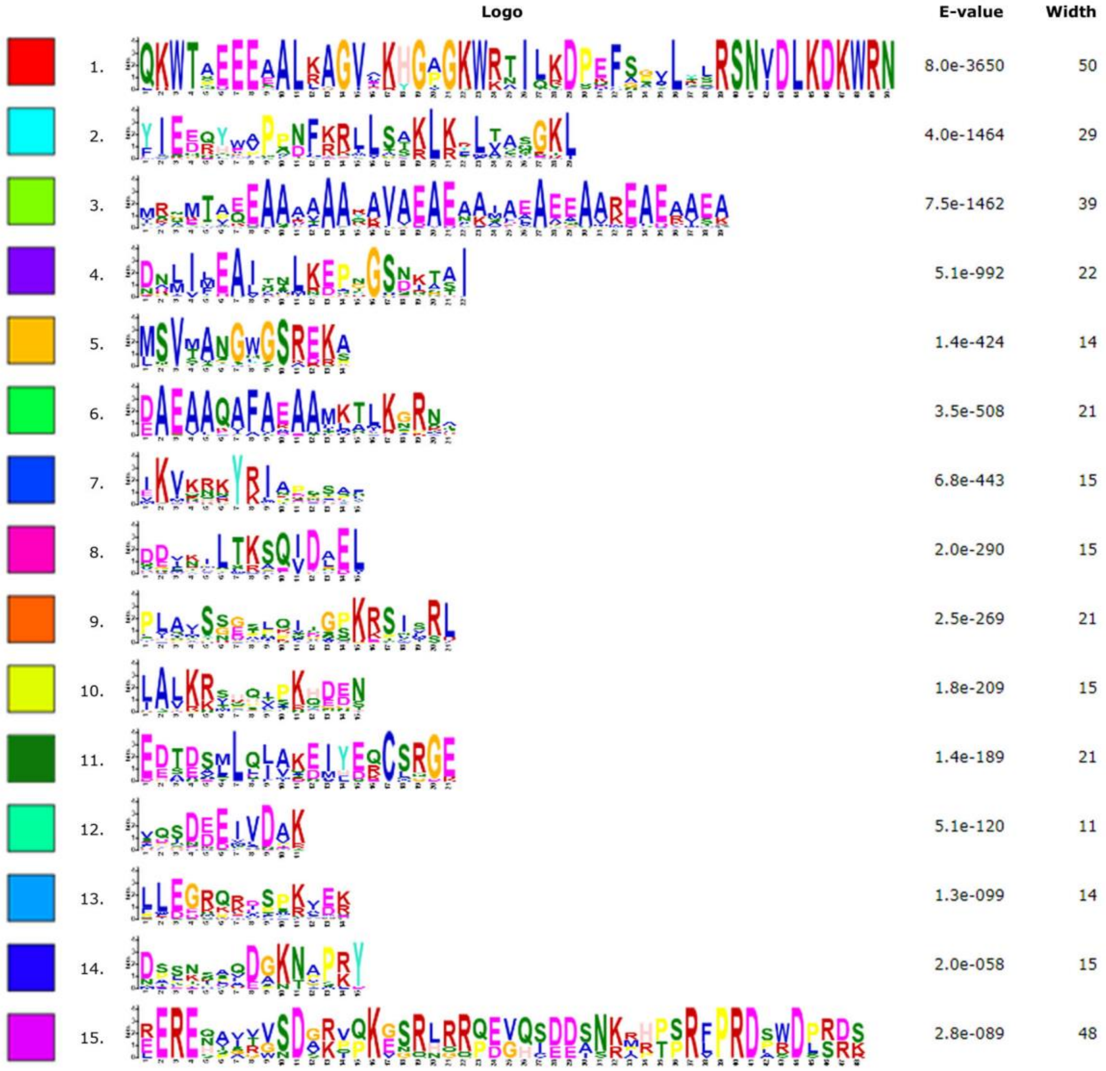

Supplemental Fig. 1 The 15 most conserved motifs in plant TRB protein motifs

Motifs are ranked and ordered by highest probability of occurrence. Motif 1 corresponds to the MYB-like domain. Motifs 2, 4 and 7 belong to the H1/H5-like domain. Motifs 3 and 6 or 3 and 11 create the coiled-coil domain.

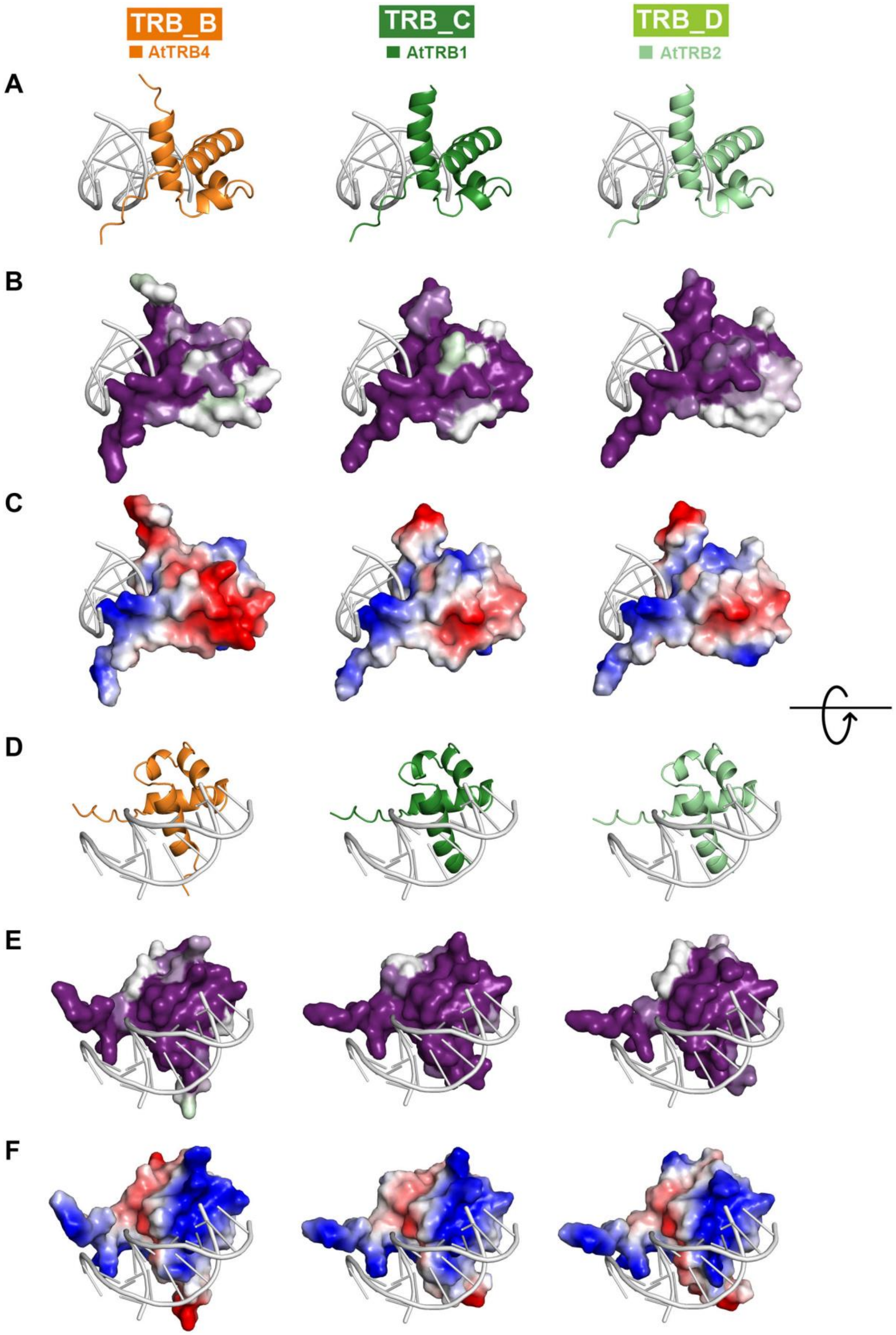
TRB_B
AtTRB4
TRB_C
AtTRB1
TRB_D
AtTRB2
A
B
C
D
E
F

Supplemental Fig. 2 Conserved residues and electrostatic charge visualization of the Myb-like domain in *Arabidopsis* members of Dicots from TRB_B, TRB_C and TRB_D lineages

The representative members of Dicots from TRB_B, TRB_C and TRB_D lineages, namely *A. thaliana* TRB4, TRB1 and TRB2, respectively, were analyzed. The three-dimensional model of the Myb-like domains from the site opposing the DNA-binding (A-C) and from the DNA binding (D-F) viewpoints are based on the hTRF2-DNA interaction model (PDB: 1WOU) (Court et al. 2005).

B) and E) The evolutionary dynamics of aa substitutions among aa residues were visualized in ConSurf 2016 (Ashkenazy et al., 2016). The conservation of residues is presented in a scale, where the most conserved residues are shown in dark magenta and non-conserved residues as white.

C) and F) Surface models showing the charge on the Myb-like domains. Residue charges are coded as red for acidic, blue for basic, and white for neutral, visualized using PyMol, Version 2.4.1, Schrödinger, LLC.

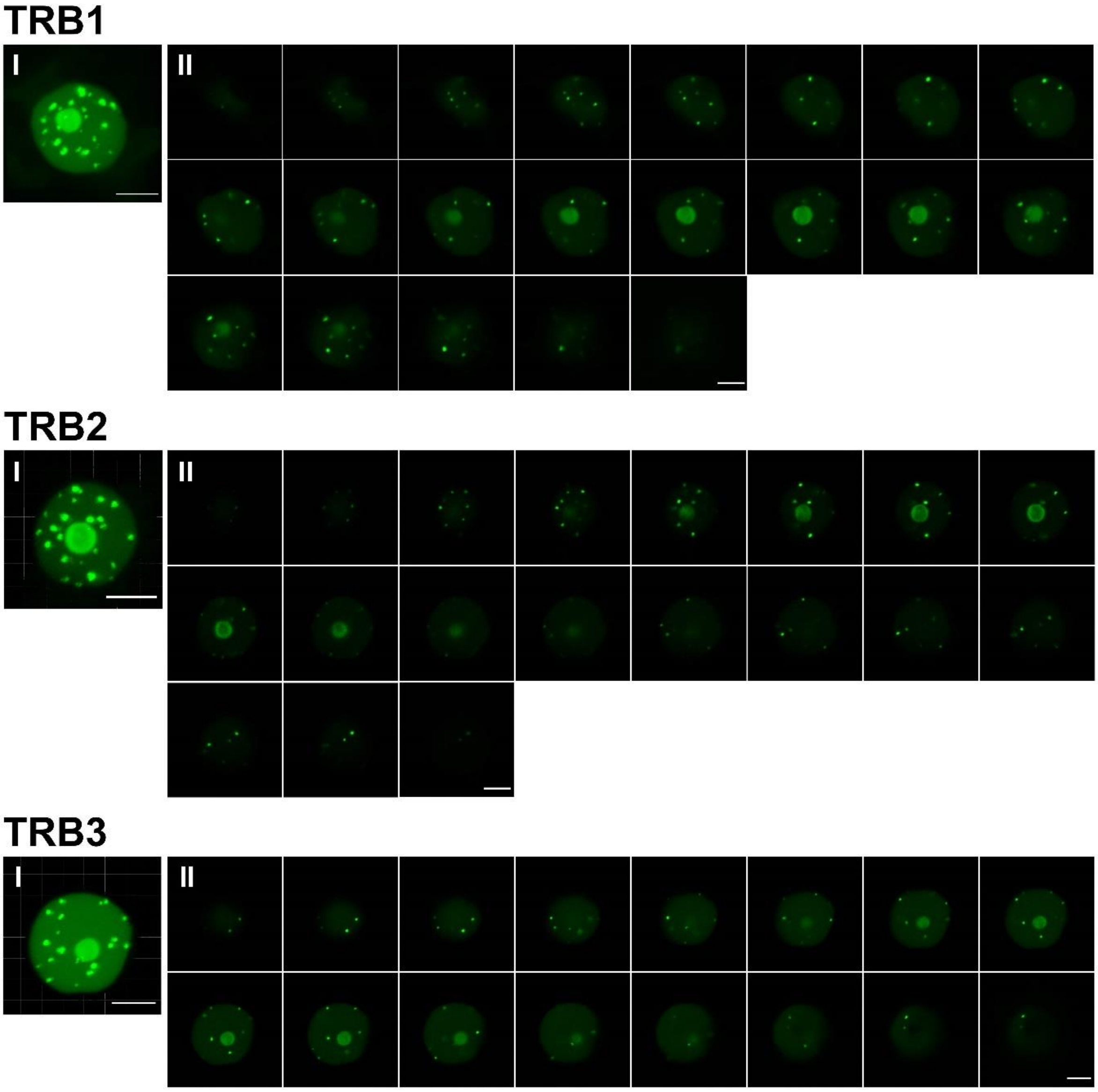

Supplemental Fig. 3 Maximum intensity projections and Z-stacks of TRB1-3 GFP-fusion proteins in nuclei of *N. benthamiana* leaf epidermal cells

TRB1-3 fused with GFP (N-terminal fusions), expressed in *N. benthamiana* leaf epidermal cells and observed by confocal microscopy. Figures represent Maximum Intensity projections (I) of entire Z-stack images of nuclei (II; TRB1 - 0,53 µm each; TRB2 and TRB3 – 0,47 µm each). Scale bars = 5 µm.

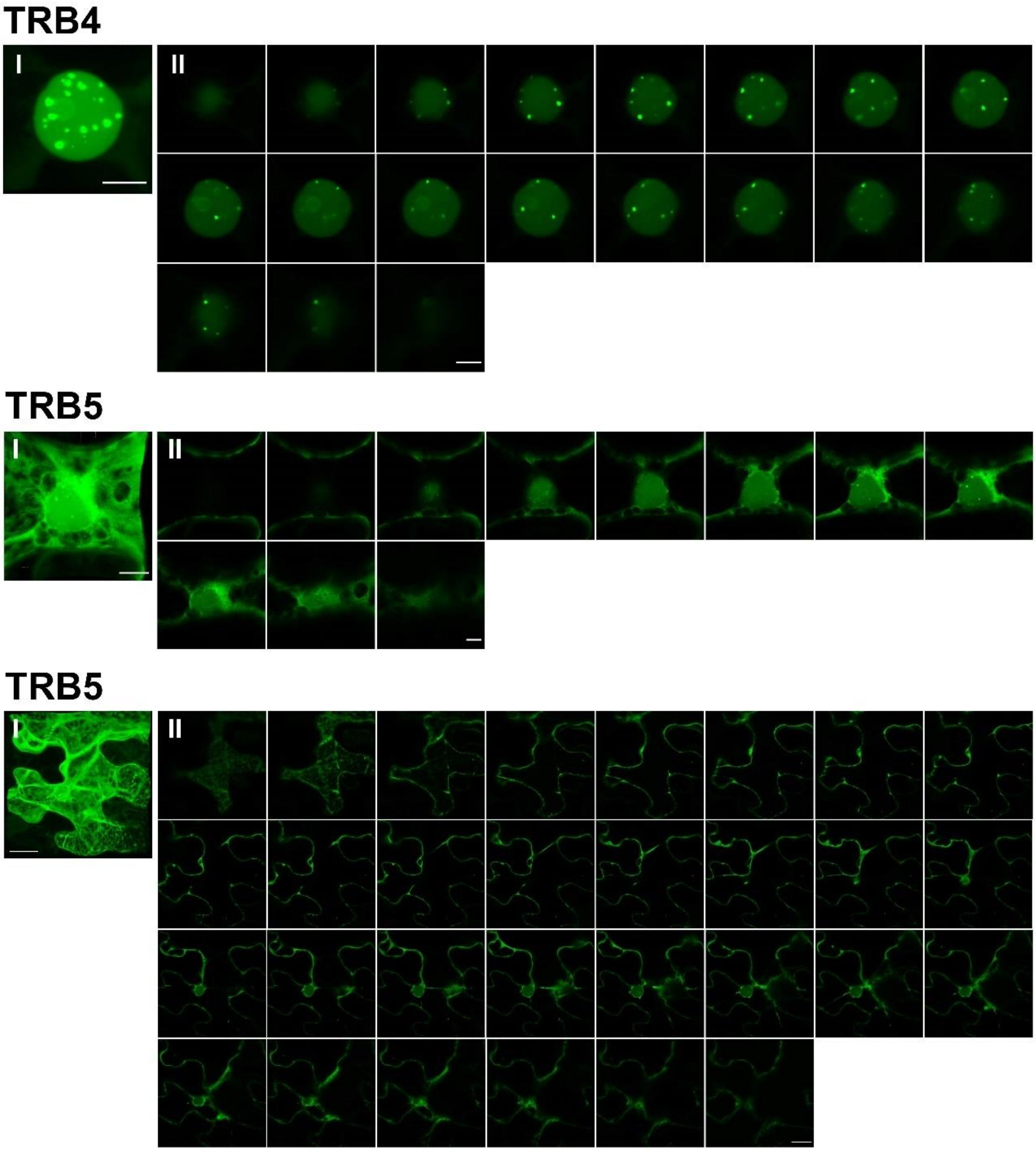

Supplemental Fig. 4 Maximum intensity projections and Z-stacks of TRB4-5 GFP-fusion proteins in *N. benthamiana* leaf epidermal cells

TRB4-5 fused with GFP (N-terminal fusions), expressed in *N. benthamiana* epidermal cells, and observed by confocal microscopy. Figures represent Maximum Intensity projections (I) of entire Z-stack images (II) of nuclei (TRB4 - 0,52 µm each; TRB5 - 0,82 µm each; scale bars = 5 µm) and whole epidermal cell (TRB5 - 0,78 µm each; scale bar = 20 µm).

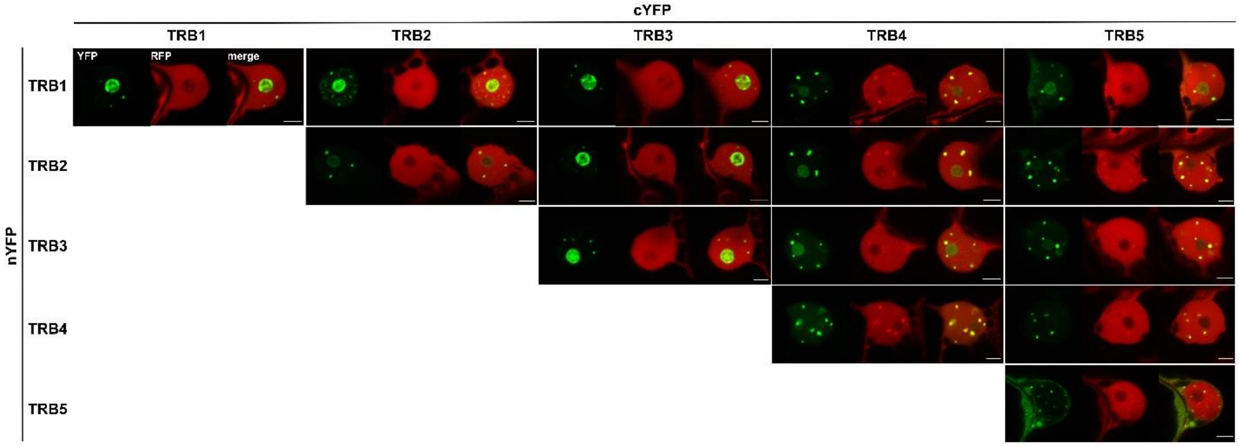

Supplemental Fig. 5 Mutual TRB interactions detected by BiFC in *N. benthamiana*

Protein-protein interactions of TRB proteins fused with nYFP or cYFP part were detected in *N. benthamiana* leaf epidermal cells by confocal microscopy. Shown here are single images of fluorescence signals from individual channels (*YFP*, Yellow fluorescence protein; *RFP*, red fluorescence protein – an internal marker for transformation and expression) and merged signals (*merge*, merged YFP and RFP channels). Scale bars = 5 µm.

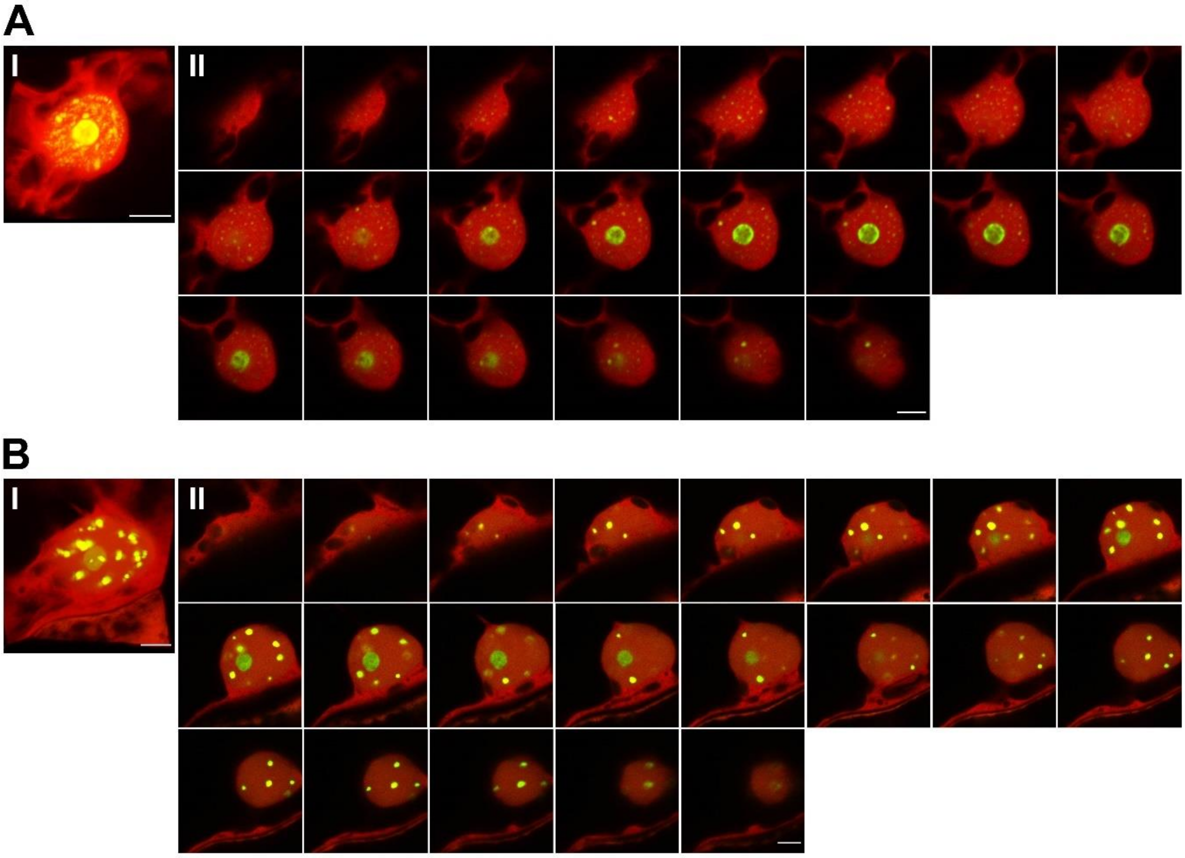

Supplemental Fig. 6 Maximum Intensity projections and Z-stacks of *N. benthamiana* epidermal cells nuclei presenting BiFC analyses

Maximum Intensity Projections (I) of entire Z-stack images (II) of nuclei of *N. benthamiana* leaf epidermal cells displaying BiFC interactions of TRB proteins. Shown here are merged images of YFP (interaction of the tested proteins) and mRFP (internal marker for transformation and expression) fluorescence detected by confocal microscopy. Scale bars = 5 µm.

A) Interaction of TRB1 and TRB2 proteins (nYFP-TRB1 + cYFP-TRB2 pBiFCt-2in1-NN construct). Optical sections 0,48 µm each.
B) Interaction of TRB1 and TRB4 proteins (nYFP TRB1 + cYFP TRB4 pBiFCt-2in1-NN construct). Optical sections 0,55 µm each.

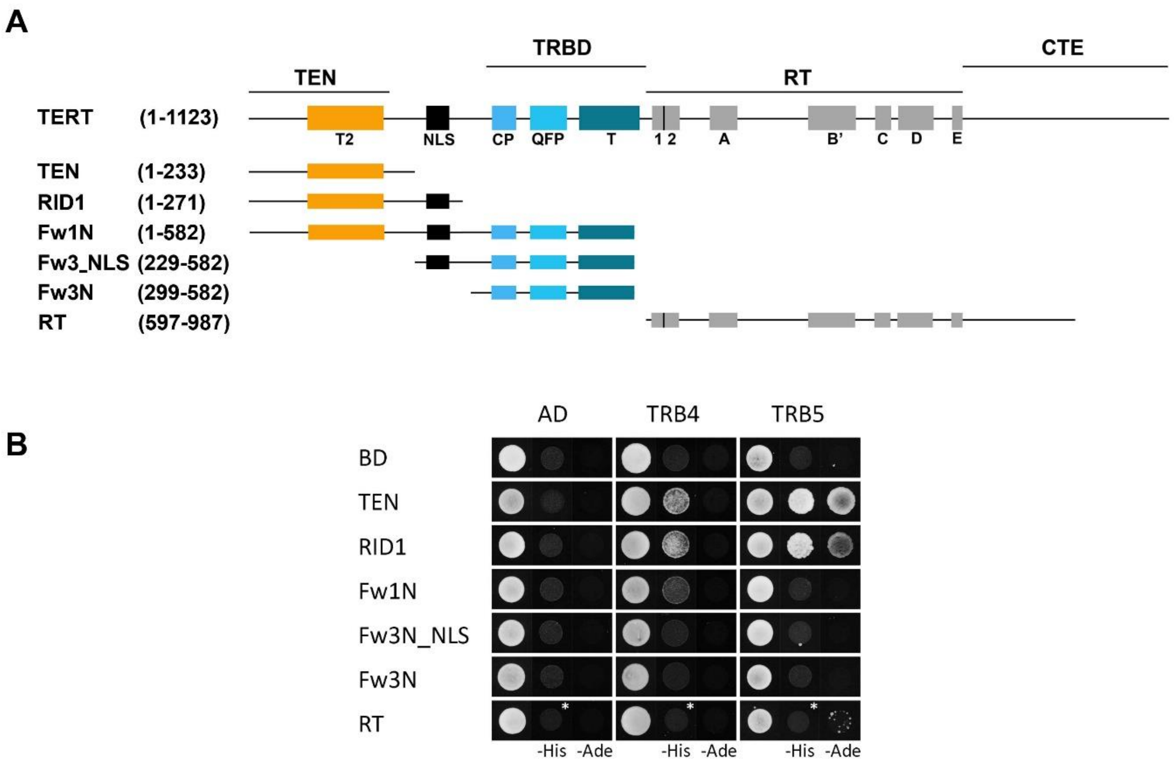

Supplemental Fig. 7 Interactions of TRB4-5 with TERT domains

A) Schematic depiction of the plant catalytic subunit of telomerase (TERT) showing functional motifs. The regions of structural domains TEN (telomerase essential N-terminal domain), TRBD (Telomerase RNA-binding domain), RT (reverse transcriptase domain) and CTE (C-terminal extension) are depicted above the conserved RT motifs (1, 2, A, B, C, D and E), telomerase-specific motifs (T2, CP, QFP and T) and a NLS (nucleus localization-like signal). All of the depicted TERT fragments were used for protein-protein interaction analysis (amino acid numbering is shown).

B) TERT fragments from Majerská et al. 2017 were fused with the GAL4 DNA-binding domain (BD). TRB4 and TRB5 were fused with the GAL4 activation domain (AD). Both constructs were introduced into yeast strain PJ69-4a carrying reporter genes His3 and Ade2. Interactions were detected on histidine-deficient SD medium (-His), or under stringent adenine-deficient SD medium (-Ade) selection. Co-transformation with an empty vector (AD, BD) served as a negative control. *Asterisks* *, 3 mM 3-aminotriazol.

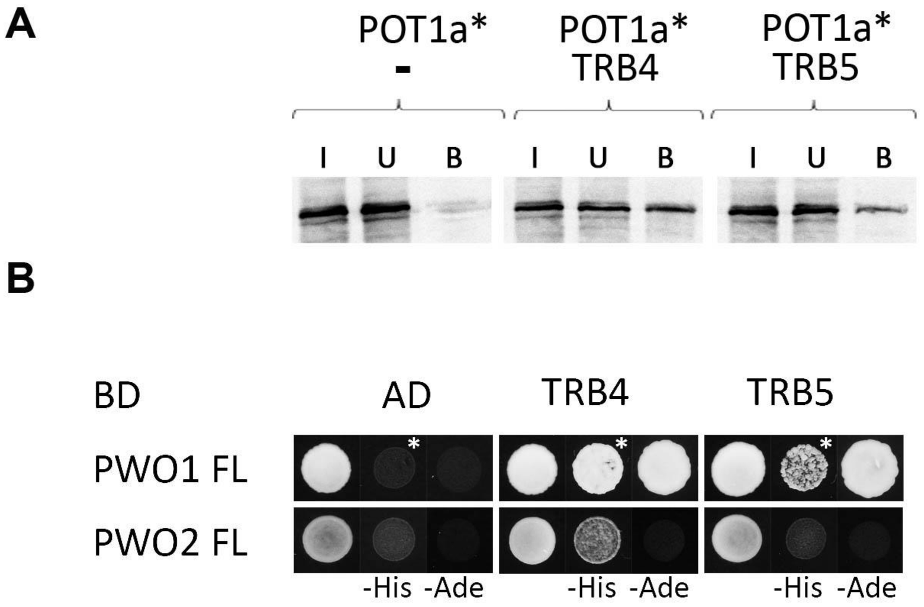

Supplemental Fig. 8 Protein-protein interactions of TRBs with various proteins

A) TNT expressed proteins, POT1a (^{35}S-labelled*) and TRB4/TRB5 (myc-tag), were mixed and incubated with an anti-myc antibody and Protein G magnetic particles. In the control experiment, the POT1a proteins were incubated with an anti-myc antibody and Protein G magnetic particles in the absence of partner protein. Input (I), unbound (U), and bound (B) fractions were collected and run in SDS–10% PAGE gels. *Asterisks* *, ^{35}S labelling.

B) Interactions of TRB4-5 were evaluated using the Y2H system. Interactions between TRB4-5 and full-length PWO1-2 were tested as in Fig. 7B. Interactions were detected on histidine-deficient SD medium (-His), or under stringent adenine-deficient SD medium (-Ade) selection. Co-transformation with an empty vector (AD, BD) served as a negative control. *Asterisks* *, 3 mM 3-aminotriazol.
